# Supplementary material for: Abiotic processes control carbon dioxide dynamics in temperate karst lakes
Source: PeerJ. 2024 May 22;12:e17393. doi: 10.7717/peerj.17393 (PMC11127641; doi:10.7717/peerj.17393)
Supplement: Supplemental Information 1 [file peerj-12-17393-s001.docx]

| **Table S1.** Location and main characteristics of the studied lakes. Lat = latitude, Long = longitude, Alt = altitude, Vol = volume, Z_MAX_ = maximum depth, Z_M_ = mean depth. | | | | | | | | | |
| --- | --- | --- | --- | --- | --- | --- | --- | --- | --- |
| **Lake** | **Lat.** | **Long.** | **Alt.** | **Vol.** | **Surface area** | **Z_MAX_** | **Z_MEAN_** | **Residence**  **time** | **Urban area** |
|  | **(N)** | **(W)** | **(m a.s.l.)** | **(hm^3^)** | **(ha)** | **(m)** | **(m)** | **(y)** | **(km^2^)** |
| Conceja (LC) | 38.9211 | 2.8097 | 863 | 2.4 | 29 | 14 | 8.4 | 4.1 | 0.0004 |
| Colgada (CG) | 38.9606 | 2.8922 | 799 | 8.6 | 100 | 18 | 9.8 | 18.4 | 1.98 |
| Cueva Morenilla (CM) | 38.9841 | 2.9158 | 772 | 0.4 | 7 | 8 | 5.5 | 13.6 | 2.37 |

**Table S2.** Averages (± standard deviation) of water temperature (ºC), dissolved oxygen (DO), pH, electric conductivity (K_25_), and euphotic zone length (Z_EU_) in Conceja Lake (LC), Colgada Lake (CG), and Cueva Morenilla Lake (CM) during summer and winter. Based on KW results, season (capital letters) and lakes (lowercase letters) were separated using the Dunn pairwise comparison test, similar letters are not different at α=0.05. [Sig. Diff: significant differences].

| **Season** | **Lake** | **n** | **T** | **DO** | **pH** | **K_25_** | **Z_EU_** |
| --- | --- | --- | --- | --- | --- | --- | --- |
|  |  |  | **(ºC)** | **(mg L^-1^)** |  | **(µS cm^-1^)** | **(m)** |
| Summer |  | 27 | 21.8 ± 2.3*^A^* | 10.6 ± 2.6*^A^* | 7.8 ± 0.2*^A^* | 666 ± 5*^A^* | 27.3 ± 10.2*^A^* |
|  | LC | 9 | 20.0 ± 2.3*^abc^* | 12.7 ± 3.5*^a^* | 7.7 ± 0.1*^ab^* | 693 ± 70*^ab^* | 22.7 ± 6.5*^a^* |
|  | CG | 9 | 21.9 ± 2.0*^ab^* | 10.5 ± 0.9 *^ab^* | 7.5 ± 0.2*^a^* | 680 ± 28*^ab^* | 36.3 ± 10.6*^a^* |
|  | CM | 9 | 23.4 ± 0.9*^a^* | 8.7 ± 0.8*^b^* | 8.0 ± 0.03*^bc^* | 626 ± 2*^a^* | 22.9 ± 7.5*^a^* |
| Winter |  | 18 | 10.0 ± 0.7 *^B^* | 11.8 ± 0.5*^B^* | 8.2 ± 0.2*^B^* | 691 ± 46*^B^* | 33.3 ± 7.0*^B^* |
|  | LC | 6 | 9.8 ± 0.5*^cd^* | 11.6 ± 0.2*^a^* | 8.1 ± 0.02*^c^* | 754 ± 3*^b^* | 38.2 ± 5.5*^a^* |
|  | CG | 6 | 10.5 ± 0.8*^bcd^* | 12.2 ± 0.5*^a^* | 8.1 ± 0.3*^bc^* | 665 ± 5*^ab^* | 35.4 ± 1.9*^a^* |
|  | CM | 6 | 9.6 ± 0.4*^d^* | 11.4 ± 0.5*^a^* | 8.3 ± 0.01*^c^* | 656 ± 7*^a^* | 26.2 ± 6.4*^a^* |
